# Outcome of an Experimental Study in Growing Turkeys Suspected of Having a Diet Related, Uncommon and Uncoordinated Gait

**DOI:** 10.3390/vetsci4040049

**Published:** 2017-09-30

**Authors:** Amr Abd El-Wahab, Christian Visscher, Christine Ratert, Mareike Kölln, Daniel Diephaus, Andrease Beineke, Josef Kamphues

**Affiliations:** 1 Department of Nutrition and Nutritional Deficiency Diseases, Faculty of Veterinary Medicine, Mansoura University, Mansoura 35516, Egypt; amrabdelwahab37@yahoo.de; 2 Institute for Animal Nutrition, University of Veterinary Medicine Hannover, Foundation, Hannover 30173, Germany; christian.visscher@tiho-hannover.de (C.V.); christine.ratert@tiho-hannover.de (C.R.); mareike.koelln@tiho-hannover.de (M.K.); 3 Tierarztpraxis Am Dorfteich, Cloppenburg 49661, Germany; dd@tierarztpraxis-am-dorfteich.de; 4 Institute for Pathology, University of Veterinary Medicine Hannover, Foundation, 30559, Germany; andreas.beineke@tiho-hannover.de

**Keywords:** abnormal gait, vitamin A, turkeys

## Abstract

On the occasion of a clinical case on a turkey farm and based on the suspicion that the diet composition could be the cause, an eight-week diagnostic trial was performed with turkey poults (n = 54) divided into two groups (control and experimental). The levels of vitamin A in the starter and grower diets of the control group were 7168 and 5213 IU/kg diet, but <1000 IU/kg in the experimental ones. Vitamin A and uric acid contents were measured in the serum, while liver samples were taken to determine the vitamin A content. Parts of the central nervous system and some internal organs were examined histologically. In the sixth week, ruffled feathers and uncoordinated gait were the earliest signs seen in the experimental group. The vitamin A content in the liver samples significantly decreased in the experimental group (0.09 mg/kg vs. 29.5 mg/kg). The serum level of uric acid in the experimental group was significantly higher (12.8 mg/dL vs. 3.38 mg/dL). Birds in the experimental group showed squamous metaplasia in the oesophagus. No histopathological alterations were seen in the central nervous system. The elevated uric acid level in the serum is worth mentioning.

## 1. Introduction

The main challenge facing veterinary practitioners in cases of obvious clinical problems in intensive poultry production is to find out the primary cause thereof in order to treat suffering animals effectively and avoid the spread of infectious diseases. Regarding potential reasons, there is in general a need for differentiation between an infection, poisoning, mismanagement, deficiency and excess of dietary constituents like additives and technical accidents. These all might result in more or less identical disturbances, i.e., a high prevalence of animals affected. In a unit for fattening turkeys in Germany in the year 2016, it was observed by the farmers that the turkeys in the seventh/eighth week of age looked dissimilar to healthy ones, appeared “sad”/tired and the feathers were reported as being “ruffled”.

Diverse bacteriological tests were performed and some results (*Clostridia*, *E. coli*) were discussed as being the cause of disease. Nonetheless, the symptoms seemed to be non-typical. In a more detailed investigation the veterinary practitioner described an “uncoordinated gait” as the most interesting predominant finding. On one further farm—using a compound feed from the same feed plant—similar findings were reported, including uncoordinated gait. Thus, there was the suspicion that the turkeys might be affected by “blindness”, reflected in their uncoordinated gait in the stable. This, however, was not confirmed during clinical tests. During a joint meeting with the farmers, feed supplier and veterinarian, the hypothesis was made that the health problems might be caused by a vitamin A deficiency, as according to the literature feeding a vitamin A-deficient diet to poultry may result in clinical signs that include emaciation, stunted growth, unsteady gait, increased lacrimation and nasal discharge [1].

Nevertheless, other symptoms (uncoordinated gait without visual impairment) could not be explained by this theory. Other typical symptoms for a vitamin A deficiency were missing (e.g., lacrimation, blindness). Some nutritionists did not follow the hypothesis of a vitamin A deficiency because of the time frame: Up to the seventh week of age it has to be expected that at least three different batches of compound feeds (one for phase 1 = starter; two for phase 2 = grower) were fed to the animals of affected farms. It was supposed that different vitamins had been added to one premix. Thus, only vitamin E was analysed, showing expected values. It was concluded that an adequate dose of further vitamins should be present. Even in the case of a vitamin A deficiency in one feed delivery turkeys should not be affected due to vitamin A stores (in the liver) and the “short” feeding period. As different analyses on diet composition (mycotoxins, coccidiostats, misdosing of trace elements) did not result in an explanation for the observed clinically obvious symptoms, it was decided to perform a “diagnostic feeding study” in a controlled environment to rule out diseases or other influencing factors than the diet. For the experimental study remaining amounts of two diets (phase 1/phase 2) from one affected farm were available, allowing a feeding trial to commence with one-day-old poults over a period of eight weeks. The questions relating to this diagnostic study were whether and how a distinct diet from the field could be the cause of abnormal gait of growing turkeys.

## 2. Materials and Methods

The diagnostic feeding trial was performed in accordance with the German Animal Welfare Act and was approved by the Ethics Committee of Lower Saxony for Care and Use of Laboratory Animals LAVES (Niedersächsisches Landesamt für Verbraucherschutz und Lebensmittelsicherheit; reference: No. 89A163). It has to be underlined that such diagnostic feeding trials are only allowed as they are considered to be the “last chance” for an etiological elucidation. In the control group it was decided to use starter and grower diets from a farm without any clinical problems, whereas in the experimental group both diets were the suspicious ones offered at the affected fattening units. All offered diets were produced by the same company.

### 2.1. Animals and Housing

Fifty-four one-day-old turkey poults (♀, BUT-Big 6) were obtained from a commercial hatchery. At day zero, six birds were sacrificed to collect blood as well as liver samples to determine vitamin A. The remaining birds (n = 48) were randomly housed in two identical floor pens (1.50 m × 1.32 m) littered with wood shavings. The pens were equipped with a heat lamp to achieve a temperature at the outset of about 34–36 °C. The temperature was lowered by about 1 °C every two days, reaching a minimum temperature of about 20 °C.

### 2.2. Diets

The diets (henceforth referred to as experimental and control diet) were obtained from two different farms, with and without the history of affected birds (abnormal gait). Both diets were identical in all feed materials according to the labelled composition. All diets were commercially produced (either control or experimental) and based on wheat, corn, soybean meal, rapeseed meal, oil, calcium carbonate, monocalcium phosphate and sodium chloride. Both compound feeds were offered in circular troughs and water in bell drinkers. Birds were fed starter diets for the first four weeks of age, the grower diets being offered thereafter until the end of the 8th week of age. Samples of all diets were analysed (Table 1) using standardised laboratory methods [2] (with the institute’s own modifications). 

### 2.3. Experimental Design and Sampling

Individual body weight (BW) was recorded weekly to calculate weight gain during the whole lifetime. Feed intake and water consumption were measured daily at group level. Feed conversion ratio (FCR) was calculated on the basis of feed consumed throughout the experimental period as well as on body weight gain of the birds. Mortality was documented daily and considered concerning the performance parameter. The post-mortem examination and sample collection (six birds per group randomly chosen) were carried out bi-weekly throughout the entire trial. Every second week, blood samples were collected from six culled birds (according to EU directive 1099/2009) per group to determine uric acid concentration. In addition, liver samples were taken to determine the weight as well as the vitamin A concentration. Furthermore, the brain, cerebrum, cerebellum as well as spinal cord were collected, being examined both macroscopically and histologically. In addition, samples from the esophagus, proventriculus, kidneys, sciatic nerve, pectoral muscle and bursa of Fabricius were taken for histological investigations.

### 2.4. Determining Moisture Contents in Fresh Excreta and Litter Samples

To determine the dry matter and moisture content of fresh faecal and litter samples, these were collected from each pen once a week by placing a pond liner in each pen for ~one hour until about 80 g fresh pure excreta per pen had been obtained. The collected droppings were then removed from each pen, thoroughly mixed and dried at 103 °C.

Litter samples for measuring dry matter content were collected weekly from three sites (at two corners and the middle of the pen), then thoroughly mixed. Subsamples of about 100 g were taken to assess moisture content by drying at 103 °C until a constant mass was obtained. At the end of the trial, a sample from whole litter material (wood shavings, excreta, feathers) was taken from each group to determine its moisture content.

### 2.5. Foot Pad Scoring Criteria

External assessment of foot pads was performed weekly. Only the central plantar area of the foot pad was scored. Scores of foot pad dermatitis (FPD) were recorded on an eight-point scale (0 = health skin; 7 = over half of the foot pad is covered with necrotic scales) in accordance with Mayne et al. [3].

### 2.6. Vitamin A and Uric Acid Determinations

Part of the liver was taken to determine the concentration of vitamin A [4] (modified). Briefly, about 2 g of liver sample were mixed with 0.5 g ascorbic acid in a centrifuge glass tube (50 mL). Then, a mixture of 5 mL KOH and methanol (375 g KOH, 750 mL distilled water and 450 mL methanol) was added to each tube. The samples were heated for 60 min at 80 °C in a water bath (shaking the tubes every 10 min). Afterwards, the tubes were washed with cold water. Thereafter, 10 mL hexan (high-performance liquid chromatography-grade 0.1% butylated hydroxytoluene) was added to each tube. Each tube was shaken for about 10 min and then centrifuged (3000 rpm) for 2 min.

The supernatant was decanted into a 50 mL tube and the steps from adding 10 mL of hexan onwards were repeated three times. The tubes with only supernatant liquid in them were ready for extraction using hexane extractor. Ten mL of methanol (0.2 mg/L butylated hydroxytoluene) were used. The concentration of vitamin A was measured by means of liquid chromatography (CBM-20 A, Shimadzu, Kyoto, Japan). Moreover, the concentrations of uric acid in the serum of all birds were measured individually using commercial kits (Roche Diagnostics GmbH, Sandhofer Street 116, D-68305 Mannheim, Germany, Reference number: 11875426 216). Both vitamin A and uric acid concentrations were determined in the serum bi-weekly.

### 2.7. Histopathology

After evisceration, the whole skull was immersed in neutral 10% formalin. After removing and replacing the eye in neutral 10% formalin, a vertical incision was made into the skull. The brain, cerebrum, cerebellum and spinal cord were dissected free from bone and examined both macroscopically and histologically. Furthermore, parts of the oesophagus, proventriculus, kidneys, sciatic nerve, pectoral muscle and bursa of Fabricius were placed in neutral 10% formalin for examining histologically.

It has to be emphasised that only at the end of the trial the skulls of the sacrificed birds were examined radiographically.

### 2.8. Statistical Analyses

The statistical analyses were performed using the Statistical Analysis System for Windows SAS^®^, version 9.3, (SAS Institute Inc., Cary, NC, USA).

For normal distributed data, a pair-wise comparison was made for independent samples using the *t*-test. In the case of non-normally distributed data, differences in parameters between groups were assessed by using the Wilcoxon signed-rank test. All statements of statistical significance are based upon *p*-values smaller than 0.05. This approach includes the comparison-wise error rate.

## 3. Results

In the control group, no clinical signs were observed in the birds during the entire trial. One animal in the experimental group died during its second week of age.

### 3.1. Clinically Obvious Signs in the Experimental Group 

**Figure 1:**
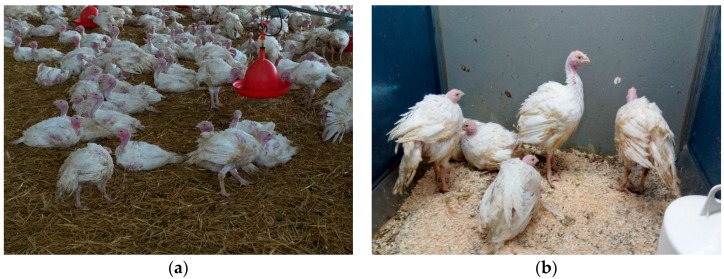
(**a**) Turkeys on a commercial farm with ruffled feathers, an inability to stand, abnormal gait. (**b**) Turkeys in the diagnostic trial were drowsy and moved uncoordinatedly when agitated, with a droopy head, ruffled feathers, undeveloped feather tail.

Primarily, it has to be stated that during the first 40 days of age there were no marked differences between the groups regarding behaviour, performance and appearance at veterinary inspection. At the beginning of the sixth week of age, an increasing number of birds of the experimental group had ruffled feathers and were lethargic, unwilling to move and showing a droopy head (Figure 1a,b).

Birds were examined several times a day by a veterinarian. The general condition of the birds was never disturbed significantly. Thus, medical treatment or stopping the trial was unnecessary. Further details regarding the reported clinical symptoms in the experimental group during this study are given below:

Days 1–40: No marked differences in movement, behaviour, posture of legs, wings, head/neck were noted. All birds seemed to be normal without any abnormal signs.

Days 42–45: The birds (50%) had ruffled feathers and the birds seemed to be “sad”, lethargic, unwilling to move with a droopy head resting on the neck. However, they were still able to go to the feeders and drinkers.

Day 48: The birds (75%) were standing in a sleeping position and, when stimulated, tried to move one or two steps forward but lost their balance when trying to put one leg to the side and the other leg backward to avoid falling. Ruffled feathers were markedly obvious.

Day 51: When the birds were provoked, they could not walk in one direction. All birds were sitting down with no will to move. Nearly all of the tail feathers had been shed. As the condition progressed they appeared very drowsy and only moved when attacked.

Day 54: Although the birds were sitting most of the time, they were still able to go to the troughs and drinkers with some difficulty. The wings appeared to be hanging down on the litter.

Generally, throughout the trial there were no cases of body tremors or blindness or birds that only lay in a lateral position. No evidence of lacrimation or nasal discharge was seen, as described in the literature.

### 3.2. Diet, Performance, Side Effects

The complete data of both diets are listed in Table 1. The nutrient contents of both diets were almost identical in starter or grower phases except for vitamin A content. The levels of vitamin A in the starter and grower diets of the control group were 7168 and 5213 IU/kg diet, respectively, but <1000 IU/kg in the experimental ones.

Data in Table 2 show that birds fed control diets (starter/grower) had a higher total feed intake (77.2 kg) than those fed the experimental diets (73.1 kg). Nevertheless, birds fed the experimental diet had a higher total water intake (217 kg) than those fed the control diet (197 kg). Thus, birds fed the experimental diet had a higher total water:feed intake ratio in comparison to those fed the control diet (2.96 vs. 2.55).

Table 3 shows the development of BW for turkeys throughout the trial. No significant differences in BW (3839 g ± 430 for control group vs. 3712 g ± 444 for experimental group) were observed between both groups from the 1st week of age until the end of the trial. 

Table 4 shows the dry matter content of both excreta and litter. Feeding experimental diet resulted in numerically lower dry matter content of excreta (on average during the entire trial: 15.5% vs. 19.2%). Birds fed control diet had numerically higher litter dry matter content (61.3%) than those fed experimental diet (46.6%). Furthermore, the dry matter content of the whole final litter was markedly lower (37.4%) in the group fed the experimental diet than in the group fed the control diet (65.3%). FPD scores of birds are shown in Table 4. Significant differences were observed in FPD scores from the 2nd to the 8th week of age. Birds fed the experimental diets had significantly higher FPD scores than those fed the control diet (6.9 vs. 3.1 at end of trial). Concerning the prevalence of FPD scores at the 8th week of age, 83.3% birds fed the control diet had low scores (0–3) and 16.7% medium scores (3.5–5), while 100% birds fed the experimental diet had high/severe FPD scores (5.5–7).

### 3.3. Liver Weight and Vitamin A Content 

No significant differences were observed in the liver weight between birds fed the control diet and those fed the experimental one (Table 5). Moreover, the liver weight increased by an average of 67 g from the 2nd week till the 8th week of age for all birds regardless of whether they had been fed the control or experimental diets. The level of vitamin A in the liver of the six sacrificed one-day-old poults was 29.7 ± 6.7 mg/kg (fresh basis). Liver weight as well as vitamin A concentrations in the liver are shown in Table 5. Significant differences were found between birds fed the control diet and those fed the experimental diet during the whole trial. After only two weeks of age, the vitamin A content in the liver was significantly higher than for those birds fed the experimental diet (17.9 ± 3.20 vs. 1.81 ± 1.13 mg/kg).

### 3.4. Serum Parameters

Results of vitamin A concentrations in the serum are shown in Table 5. Level of vitamin A in serum varied at 1.1 ± 0.2 mg/L of one-day-old birds. Only after two weeks of age the birds fed the experimental diet had a lower concentration of vitamin A in the serum than those fed the control diet (0.466 ± 0.054 mg/L vs. 1.07 ± 0.167 mg/L). The concentration of vitamin A in the serum of birds fed the control diet varied at a constant level during the entire trial (1.1 mg/L) whereas in the experimental group the values declined to about 20% of normal values. 

Concomitantly, the uric acid concentration in the serum of birds fed the experimental diet had significantly higher values than those fed the control diet (Table 5). After the second week of age, birds fed the experimental diet had 9.91 ± 2.14 mg uric acid/dL serum. Additionally, at the eighth week of age the level of uric acid in the serum of experimental birds was significantly higher (12.8 ± 3.21 mg/dL) than those observed in the control group (3.38 ± 0.847 mg/dL).

### 3.5. Histopathological Findings

At post-mortem examination, no obvious histological alterations were seen in the oesophagus, proventriculus and bursa of Fabricius throughout the whole trial in the control group. Moreover, no macroscopical or histopathological alterations were found in the eye, sciatic nerve, cerebrum, cerebellum and spinal cord and pectoral muscle of the birds fed either normal or vitamin A-deficient diets. Also, there was no evidence of central nervous system (CNS) compression or herniation in the histopathological or radiographic examinations at the end of the trial. 

**Figure 2:**
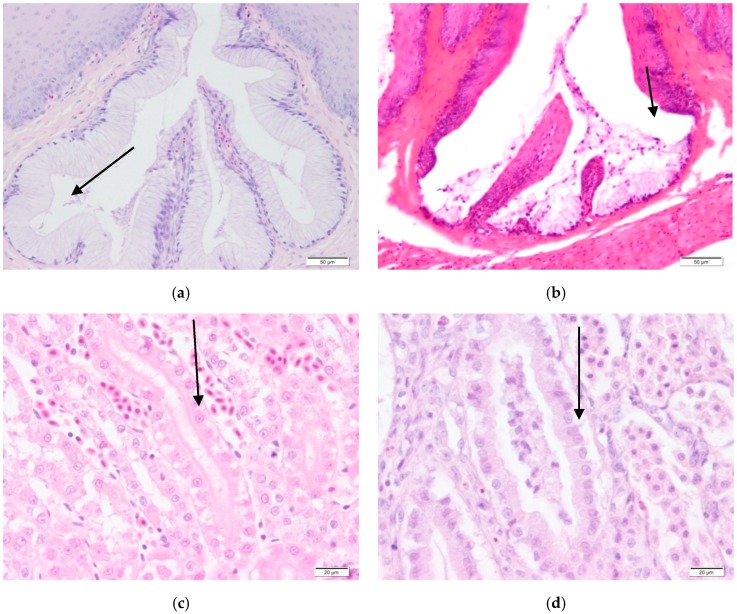
Microscopic lesions of tissues from control (**a,c**) and experimental birds (**b,d**). (**a**) Normal oesophageal mucosa and mucus glands (arrow). (**b**) Oesophagus with hyperplastic, thickened mucosa and submucosal squamous metaplastic mucus glands (arrow). (**c**) Normal renal tubules (arrow). (**d**) Mild degeneration in renal tubules (arrow).

However, the histopathological findings of all birds exposed to a vitamin A deficient diet revealed a squamous metaplasia in the oesophagus, proventriculus and bursa of Fabricius (Figure 2b). These mild lesions appeared after 42 days of being fed the vitamin A-deficient diet, with the lesion becoming more and more obvious after 56 days of age.

Special attention was given to kidneys as there was a mild degeneration of epithelia in the renal tubules in all deficient poults at the end of the trial (Figure 2d). Urate deposits were found in the kidneys of all vitamin A-deficient birds at the end of the trial in the birds fed vitamin A-deficient diets.

## 4. Discussion

Without any doubt, it can be stated that the vitamin A content in the experimental diet was lower than one-fifth of the recommended values (~5000 IU/kg) and resulted in marked vitamin A deficiency in the birds. In spite of mobilising the liver stores, the vitamin A levels in the serum could not be maintained. Already after two weeks the levels in the serum were reduced to 50%. Later on, alterations in the oesophagus and kidney were also found; the latter might have resulted in high serum levels of uric acid. However, it has to be underlined that it took about 6–7 weeks before further signs were provoked. Particularly, it needs to be emphasised that the “main symptoms” did not occur before weeks 6–7.

There is one main question: How to explain the “uncoordinated gait” that prompted the testing of these diets in a diagnostic feeding trial. There are some studies [5,6,7] in which “ataxia”, “uncoordination”, and “sadness” were the main signs in the affected birds fed vitamin A-free diets.

In the case of reduced uric acid excretion, it is questionable whether these high serum levels are responsible for CNS affection. In mammals, high levels of ammonia are accompanied by CNS symptoms like uncoordination. The fact that we failed to demonstrate high ammonia levels in the serum may be due to the time lapse between sampling and analysing the serum.

There is a further explanation for the “uncoordinated gait”, this being related to the role of vitamin as a “morphogen”. In vitamin A deficiency there is a risk for high pressure in the cavity containing the cerebrospinal fluid due to impaired growth and development of the skeleton including the spinal cord and lumbosacral vertebrae.

According to Hill et al. [8], about 55% of birds fed 440 IU vitamin A/kg diet at first four weeks of age showed ataxia with 25% mortality rate and body weight of 278 g. Years ago, Harms et al. [9] reported that chicks fed a vitamin A-deficient diet developed signs of vitamin A deficiency after four weeks. However, feeding a vitamin A-deficient diet for only 20 days resulted in vitamin A deficiency signs (uncoordination) in turkeys [1,6]. In our case study, signs of uncoordination as well as ruffled feathers were noted after six weeks of feeding a vitamin A-deficient diet until the end of the trial (eight weeks). In another study, listlessness and unsteady gait were also observed in earlier times in vitamin A deficiency in both chickens and poults [10].

### 4.1. Feed Intake

In previous studies [6,11] a significant decrease in feed intake in chicks and turkeys fed diets deficient in vitamin A (between 500 and 1000 IU vitamin A/kg diet) was recorded. An interesting parameter is the water intake which was markedly higher in birds fed diets deficient in vitamin A. It is known that a high water intake might be caused by either higher sodium/potassium levels in the diets or impaired intestinal absorption. Regarding the chemical analyses of the diets, no marked differences were observed for the levels of sodium and potassium in both diets. This lends support to the hypothesis of alterations in the intestinal epithelium. It is well known that efficiency of water absorption is affected by (intestinal) health. Impaired intestinal integrity reduces net water absorption from the intestinal tract causing wet litter and increased FPD scores [12].

### 4.2. Body Weight

Regarding the performance parameters, our results agreed with those of Richter et al. [11], who could not find a connection between vitamin A supplementation and body weight of the birds. In another study, the BW of broilers fed 2500 IU vitamin A/kg diet was 1590 g vs. 1553 g for those fed 10000 IU vitamin A/kg diet at eight weeks of age [13]. Also, Kuenzel et al. [7] showed that birds fed a diet that only meets half of the vitamin A requirement had a BW development comparable with control ones up until day 49.

However, in our study, the BW was numerically higher in the control group at day 56. The cause of low BW in the experimental group could be as stated by Prinz et al. [14], who found that turkeys fed 125–750 IU vitamin A/kg diet had lower growth rates in comparison to those fed 2000 IU vitamin A/kg diet.

### 4.3. Vitamin A in Liver 

In the present study, it was observed that feeding an adequate level of vitamin A in the diet was accompanied by increase in liver storage of vitamin A. Similarly, an adequate storage of vitamin A in the liver of young turkeys required about 5000 IU/kg feed [6]. Also, Harms et al. [9] observed that feeding chicks from 500 to 10,000 IU vitamin A per pound of diet produced a step-wise increase in the vitamin A content of the liver (from 8.1 to 463 IU vitamin A/g liver). Moreover, one week of feeding a vitamin A-free diet to broilers resulted in 280 IU vitamin A/g liver vs. 370 IU vitamin A/g liver for those fed a normal diet [15]. According to Whitemore et al. [16], the level of vitamin A in the liver of turkeys positively correlated to the dietary vitamin A supply as follows: 1653, 6614, 13228 and 19841 IU vitamin A/kg diet resulted in 12, 412, 2417 and 3940 IU vitamin A in the liver, respectively. Thus, Whitemore et al. [16] recommended dietary levels for vitamin A for turkeys: 15,000 (0–6 w); 10,000 (7–12 w); 7500 IU/kg (>12 w).

### 4.4. Uric Acid in Serum

A very high concentration of uric acid in the serum of birds fed a vitamin A diet was not expected after only two weeks of feeding a vitamin A-deficient diet. Presumably the uric acid metabolism is not disturbed, but the kidney undergoes definite pathological changes during severe vitamin A deficiency which might impair the normal elimination of uric acid. The concentration of uric acid in the blood is dependent upon the degree of kidney damage. Years ago, chickens fed a vitamin A-free diet from the age of one day onwards resulted in high concentrations of serum uric acid from 7 mg/dL to >20 mg/dL at seven weeks of life vs. ~5 mg/dL for those fed normal vitamin A levels [17]. Also in another study, the uric acid concentration in the serum of chicks fed a vitamin A-deficient diet increased above normal ones [18].

### 4.5. Histopathology

Among the histopathological findings, the most characteristic lesion was squamous metaplasia in the oesophagus. Similarly, metaplasia changes were found in chicks fed a vitamin A-free diet for three-four weeks [19]. Also, turkeys fed experimental vitamin A deficient diet were characterised by squamous metaplasia in the oesophagus, oropharynx, proventriculus and bursa of Fabricius [20]. Microscopically, squamous metaplasia is the most characteristic lesion of vitamin A deficiency, affecting epithelia of the digestive, respiratory and urinary tracts [1,20,21].

### 4.6. CNS

There are controversial debates regarding the extent to which changes in the CNS occur and have clinical implications. In this study, no gross/histopathological lesions were found in the CNS. Also, no lesions were seen in the brains of ataxic vitamin A deficient chickens [22]. Nevertheless, chicks fed a vitamin A deficient diet had marked changes in the CNS including herniation of the brain into venous sinuses of the head, distortion of the cerebellum and medulla and compression of the spinal cord [23]. Years ago, it was a generally accepted opinion that the changes in the CNS were caused by the direct pressure of abnormal bony excrescences and not by retarded bone growth [24]. Moreover, the CNS was indirectly affected by macroscopical and microscopical compression and high pressure of cerebrospinal fluid (212 mm) when cockerels were fed a vitamin A-free diet [5]. Furthermore, feeding a vitamin A-deficient diet to chicken resulted in a significant elevated pressure in the cerebrospinal cavity [7,25]. 

### 4.7. Side Effects

The present study was not focussed on litter quality and foot pad health [3], but the severity of FPD was clearly affected by diet and litter quality, i.e., high litter moisture is known as predominant factor in the development of FPD [26,27,28]. In the group fed vitamin A-deficient diets, a higher water intake was observed, along with very wet litter and high scores of foot pad lesions. High litter moisture could be due to impaired intestinal absorption. Another hypothesis may be that the birds tried to drink more and more water to excrete the accumulating uric acid. Furthermore, it is worthwhile underlining the percentage of the markedly high FPD scores 6–7 in the current study (~100% for birds fed vitamin A-deficient diet).

## 5. Conclusions

There is no doubt that dietary undersupply of vitamin A from the first day of age resulted in a marked vitamin A deficiency in birds, this being indicated best by the levels of vitamin A in the liver and serum. Astonishingly, about six weeks of dietary deficiency were required to induce the impaired moving capacity “imbalance”. However, the main question is the pathomechanism behind this. Regarding the role of vitamin A as a “morphogen”, it is assumed that increased pressure of cerebrospinal fluid might have an effect. Furthermore, the elevated uric acid level in the serum is worth mentioning. In other mammalian species, hyperammonia is accompanied by CNS affections (including uncoordinated gait). Considering the importance of conducting diagnostic feeding trials, some speculations such as “new infective disease” could be ruled out. During the whole trial, typical “textbook signs” did not occur. Regarding the duration of the diagnostic trial, there was no need to extend the trial period as the signs that occurred were accompanied by very low levels of vitamin A in the liver and serum.

## Figures and Tables

**Table 1:** Analysed nutrient contents of the diets during the whole trial on dry matter basis.

Nutrients	Unit	Starter (1st–4th Week of Age)		Grower (5th–8th Week of Age)	
		Control *	Experimental *	Control ^†^	Experimental ^†^
Dry matter	g/kg	889	892	887	887
Ash	g/kg	78.9	77.9	59.2	68.5
CP	g/kg	293	294	238	263
EE	g/kg	49.5	52.8	60.5	60.5
CF	g/kg	35.2	35.9	34.8	32.5
NfE	g/kg	542	537	605	573
Starch	g/kg	323	327	419	375
Sugar	g/kg	58.8	61.0	50.9	60.0
AME_N_ **	MJ/kg	12.4	12.6	13.4	13.2
Ca	g/kg	14.2	14.1	10.2	12.6
P	g/kg	10.8	10.8	7.53	8.52
Mg	g/kg	2.62	2.77	2.24	2.45
Na	g/kg	1.48	1.55	1.18	1.41
K	g/kg	12.9	12.9	10.1	11.14
S	g/kg	3.65	3.46	3.01	3.18
Cl	g/kg	1.70	1.88	2.50	2.83
Cu	mg/kg	23.5	25.1	24.3	21.9
Zn	mg/kg	119	121	111	108
Mn	mg/kg	130	130	109	130
Asp	g/kg	29.7	29.0	21.6	26.3
Thr	g/kg	10.4	10.8	9.01	9.30
Ser	g/kg	14.3	15.1	10.8	13.5
Glu	g/kg	55.5	59.4	52.0	54.9
Gly	g/kg	11.9	12.4	9.94	11.3
Ala	g/kg	12.2	13.1	10.3	11.5
Val	g/kg	13.3	13.9	11.3	11.2
Cys	g/kg	5.66	4.59	3.68	4.38
Met	g/kg	4.80	4.08	5.91	3.87
Ileu	g/kg	12.0	12.9	9.71	10.3
Leu	g/kg	21.7	23.2	17.5	18.8
Tyr	g/kg	10.2	10.5	8.03	8.57
Phe	g/kg	14.0	14.5	12.4	12.5
His	g/kg	7.52	7.88	6.31	6.54
Lys	g/kg	17.8	18.7	13.9	16.4
Arg	g/kg	20.5	21.8	14.8	17.3
Vit. A	IU/kg	7168	<1000	5213	<1000

All commercial diets, either control or experimental, were based on: wheat, corn, soybean meal, rapeseed meal, oil, calcium carbonate, monocalcium phosphate, sodium chloride. * diet containing (per kg; according to the labelling): 12 mg Cu, 57 mg Fe, 60 mg Zn, 80 mg Mn, 1.2 mg I, 0.35 mg Se, 9625 IU vitamin A, 4813 IU vitamin D3, 80 mg vitamin E, 500 FTU phytase, 1280 IU xylanases, 90 mg lasalocid–A-Sodium. **^†^** diet containing (per kg; according to the labelling): 12 mg Cu, 57 mg Fe, 60 mg Zn, 80 mg Mn, 1.2 mg I, 0.35 mg Se, 8750 IU vitamin A, 4375 IU vitamin D3, 80 mg vitamin E, 600 FTU phytase, 1280 IU xylanases, 90 mg lasalocid–A-Sodium. ** AME_N_ (per kg) = 0.1551 × %CP + 0.3431 × %EE + 0.1669 × %starch + 0.1301 × %sugar (as sucrose).

**Table 2:** Intake of feed and consumption of water (kg) of birds during the trial.

Parameter	Diet	Week of Age				Total Intake, kg
		0–2 (n = 24)	2–4 (n = 18)	4–6 (n = 12)	6–8 (n = 6)	
Feed intake, kg	Control	8.51	26.4	24.8	17.5	77.2
	Experimental	7.87 *	21.2	26.4	17.7	73.1
Water consumption, kg	Control	29.5	55.9	65.5	46.5	197.4
	Experimental	31.3	65.9	70.3	49.6	217.0
Water:feed intake ratio	Control	3.47	2.11	2.64	2.66	2.55
	Experimental	3.96	3.11	2.66	2.79	2.96

* n = 23 from day 9 of age

**Table 3:** Body weight (g) of birds recorded weekly throughout the trial (mean ± SD).

Diet		Week of Age							
	0 (n = 24)	1 (n = 24)	2 (n = 24)	3 (n = 18)	4 (n = 18)	5 (n = 12)	6 (n = 12)	7 (n = 6)	8 (n = 6)
Control	61.8 ± 3.6	135.3 ^**a**^ ± 16.5	311.5 ± 36.7	611.1 ± 60.1	1046.2 ± 98.8	1534.4 ± 136.4	2181.1 ± 217	2964.5 ± 308	3839.8 ± 430.6
Experimental	62.4 ± 2.9	146.6 ^**b**^ ± 14.0	322.3 * ± 33.1	633.8 ± 44.0	1073.9 ± 71.3	1597.3 ± 106	2242.8 ± 109	3029.5 ± 134	3712.3 ± 444.5

* n = 23. **^a,b^** Means in the same column with the same superscripts are not significantly different (*p* < 0.05).

**Table 4:** Excreta and litter quality as well as FPD scores of growing turkeys throughout the trial (mean ± SD).

Substrate	Diet	Week of Age							
		1	2	3	4	5	6	7	8
Excreta, DM%	Control	18.7	19.1	24.3	18.7	17.4	17.8	18.5	18.8
	Experimental	17.0	16.7	15.1	14.5	15.3	15.1	15.3	14.8
Litter, DM%	Control	88.5	70.9	64.0	61.3	58.2	54.4	48.3	45.2
	Experimental	85.4	63.7	50.4	41.6	35.6	33.2	32.5	30.4
		n = 24	n = 24	n = 18	n = 18	n = 12	n = 12	n = 6	n = 6
FPD scores	Control	1.3 ^**a**^ ± 0.5	1.6 ^**b**^ ± 0.6	1.8 ^**a**^ ± 0.3	2.1 ^**a**^ ± 0.5	2.2 ^**a**^ ± 0.3	2.5 ^**a**^ ± 0.4	2.7 ^a^ ± 0.3	3.1 ^**a**^ ± 0.2
	Experimental	1.8 ^**b**^ ± 0.7	2.3 ^**b**,^* ± 1.2	3.0 ^**b**^ ± 0.9	4.8 ^**b**^ ± 0.7	5.3 ^**b**^ ± 0.5	5.5 ^**b**^ ± 0.3	6.5 ^**b**^ ± 0.0	6.9 ^**b**^ ± 0.2

* n = 23. **^a,b^** Means in the same column with the same superscripts are not significantly different (*p* < 0.05).

**Table 5:** Liver weight, concentration of vitamin A in liver on fresh basis as well as serum concentrations of vitamin A and uric acid in growing turkeys (mean ± SD).

Parameter	Diet	Week of Age			
		2 (n = 6)	4 (n = 6)	6 (n = 6)	8 (n = 6)
Liver					
Total weight, g	Control	8.60 ± 1.40	26.6 ± 3.90	61.1 ± 9.70	74.9 ± 10.4
	Experimental	9.60 ± 2.20 *	27.0 ± 3.60	67.2 ± 10.0	77.9 ± 24.6
Vitamin A, mg/kg	Control	17.9 ^**a**^ ± 3.20	28.0 ^**a**^ ± 2.80	23.9 ^**a**^ ± 4.6	29.5 ^**a**^ ± 7.20
	Experimental	1.81 ^**b**^ ± 1.13	0.063 ^**b**^ ± 0.023	0.038 ^**b**^ ± 0.017	0.09 ^**b**^ ± 0.101
Vitamin A mg, total amount	Control	0.152 ^**a**^ ± 0.019	0.749 ^**a**^ ± 0.154	1.44 ^**a**^ ± 0.201	2.19 ^**a**^ ± 0.585
	Experimental	0.026 ^**b**^ ± 0.009	0.002 ^**b**^ ± 0.001	0.003 ^**b**^ ± 0.001	0.006 ^**b**^ ± 0.006
Serum					
Vitamin A, mg/L	Control	1.07 ^**a**^ ± 0.167	1.08 ^**a**^ ± 0.057	1.09 ^**a**^ ± 0.036	1.11 ^**a**^ ± 0.124
	Experimental	0.466 ^**b**^ ± 0.054 *	0.217 ^**b**^ ± 0.019	0.220 ^**b**^ ± 0.009	0.222 ^**b**^ ± 0.054
Uric acid, mg/dL	Control	5.93 ^**b**^ ± 1.85	5.41 ^**b**^ ± 1.61	4.26 ^**b**^ ± 0.827	3.38 ^**b**^ ± 0.847
	Experimental	9.91 ^**a**^ ± 2.14 *	11.85 ^**a**^ ± 4.14	12.48 ^**a**^ ± 3.77	12.8 ^**a**^ ± 3.21

* n = 5. **^a,b^** Means in the same column with the same superscripts are not significantly different (*p* < 0.05).

## Abbreviations:

The following abbreviations are used in this manuscript:

BW	Body weight
FCR	Feed conversion ratio
FPD	Foot pad dermatitis
CNS	Central nervous system
